# Surgical Notes

**Published:** 1886-09

**Authors:** Ralph St. J. Perry

**Affiliations:** Surgeon St. Luke’s Hospital, Cape Mount, West Africa


### SURGICAL NOTES.

BY RALPH ST. J. PERRY, M. D.,

SURGEON ST. LUKE'S HOSPITAL, CAPE MOUNT, WEST AFRICA.

Two cases of hemorrhage from stumps have lately come under the observation of the author in his associations with Prof. Jos. W. Marsee, of Indianapolis, Ind., in which persistent hemorrhage immediately following amputations has necessitated reopening of the stumps. The amputations, one of the thigh and one of the arm, followed railroad accidents, in which there was a complete mashing of the parts amputated. The operations were performed with careful antiseptic precautions and were completely successful as far as healing was concerned, the thigh case being up and about in three weeks, and the arm case entirely healed over in ten days. In each case the operator was hastily summoned about two hours after leaving the patient because of persistent hemorrhage. Upon opening the flaps it was discovered that the bone was bleeding. Alcohol was applied to the bleeding end in anticipation of an easy stoppage of the flow, but a few minutes' trial sufficed to show that this would not produce the desired effect. Monsel's solution and other strong styptics were used with no better results. At last it was determined to plug the bone. To do this the canal was hollowed out slightly, and a wad of carbolized cat-gut (which had *not* been soaked in water) was stuffed into the cavity and allowed to remain. The pressure which was brought to bear upon the arterial canals by the swelling of the gut very effectually closed them and stopped all hemorrhage. The wound was closed, redressed antiseptically, and nothing more was heard of bleeding.

Being warned by these two cases, Dr. Marsee at once purchased a yard of heavy gut, such as is used on "base viols," which was put to soak in carbolized oil. He tells me that in future cases he will use small pieces of this heavy cord instead of wads of the smaller gut. Owing to the fact that this gut is very loosely twisted and is difficult of penetration by the carbolized oil, I have determined to try carbolized decalcified bone at the earliest opportunity. At present I am preparing some bone for this use. The bones used are the long bones of the calf; they are sawed into one-half inch lengths, boiled and scraped, then digested in benzine until all fat is removed, and last of all are thoroughly dried. The pieces are now placed in dilute hydrochloric acid and allowed to remain therein until all mineral elements are eliminated. When thoroughly decalcified they are soaked in a twenty per cent. solution of carbolic acid for twenty-four hours, taken out and dried. In drying they will shrink very perceptibly, and it is hoped enough so to afford sufficient expansion when wetted to check hemorrhage from a bone. Another advantage, theoretical as yet, is that the bone will be much more readily absorbed or converted into true bone because of its sameness of tissue with the bone. I think the bones of young animals will answer the purpose better than those of old, because of the lack of compactness of their tissues, thus affording a greater amount of shrinkage and a corresponding increase of expansion; some pieces of ivory and old bone which were used in experimenting were so dense and tough that they would not shrink enough to be of any use.

*Hydrogen peroxide*, which has lately been lauded as a substitute for the acids in the treatment of chancroids, has had a fair trial in my experience, and, in my opinion, is a miserable failure. Perhaps failure in my hands was due to inability to use it properly, but as I have used it successfully in the treatment of non-venereal suppurating sores, and under the same conditions as carbolic acid, which was an effectual remedy in my hands, I am disinclined to take upon myself any great amount of the blame for non-success. I first tried it in several cases where there was only one chancroid; in each case it failed and was supplanted by carbolic acid with full success. Finally I secured a champion case, a girl with twenty-one chancroids scattered over the vulva and inner parts of the thighs. Ten of these were treated with hydrogen peroxide every second day; the remainder were burned once with the melted crystals of carbolic acid. At the end of ten days those treated with the acid were well, while the others were only slightly improved. As no one but myself knew what treatment I was pursuing, and as neither patient nor nurses interfered with the treatment, I am inclined to believe that the peroxide had as fair a show as the acid, and that it will not effect a cure in chancroids.

*Tendon suturing*, immediately following buzz-saw injuries, is an operation of doubtful value. Cases recently encountered effectually demonstrated this fact to me. Example: a case in which the tendons of the hand were drawn across the palm, the dorsal tendons all right; the parts were cleansed, the tendons sutured and the wound closed. As usual in such cases the wound healed by granulation, considerable suppuration taking place; the tendons failed to unite, and a secondary operation was necessary after the wound had entirely healed. The next case which fell under my observation was permitted to heal over before the tendons were touched; they were then cut down upon, released from their sheaths and the surrounding cicatricial tissue and sutured after the method of Wölffer.

This last case presented an interesting point in the action of buzz-saws upon tendons. The upper half of the tendons could not be found, despite the most persistent and careful search, it evidently being torn away near its origin. Study and experiment showed that when the *edge* of the saw-tooth struck the tendon it severed it at the point of contact, but when the point struck, it embedded itself in the substance of the tendon and *pulled* on it. This sudden and forcible pulling causes the tendon to snap at the weakest point, which is near the origin or muscular end. Another element usually entering into these cases is the thickened and calloused epidermis found on the hands of the mill-hands who are the victims of the saw. The enforced rest, heat of inflammation and the moisture from discharges cause a slow desquamation, and as the skin is dead it decomposes and delays granulation. I would advise that as much of this skin be cut off with scissors and scalpel as possible at each dressing.

